# Abnormal Sphingolipid World in Inflammation Specific for Lysosomal Storage Diseases and Skin Disorders

**DOI:** 10.3390/ijms19010247

**Published:** 2018-01-15

**Authors:** Marta Moskot, Katarzyna Bocheńska, Joanna Jakóbkiewicz-Banecka, Bogdan Banecki, Magdalena Gabig-Cimińska

**Affiliations:** 1 Institute of Biochemistry and Biophysics, Polish Academy of Sciences, Laboratory of Molecular Biology, Kadki 24, 80-822 Gdańsk, Poland; marta.moskot@biol.ug.edu.pl; 2 Department of Medical Biology and Genetics, University of Gdańsk, Wita Stwosza 59, 80-308 Gdańsk, Poland; katarzyna.bochenska@phdstud.ug.edu.pl (K.B.); joanna.jakobkiewicz-banecka@biol.ug.edu.pl (J.J.-B.); 3 Department of Molecular and Cellular Biology, Intercollegiate Faculty of Biotechnology UG-MUG, Abrahama 58, 80-307 Gdańsk, Poland; banecki@biotech.ug.edu.pl

**Keywords:** sphingolipid metabolism alterations, inflammatory diseases, lysosomal storage diseases, skin disorders, treatments, workable therapies

## Abstract

Research in recent years has shown that sphingolipids are essential signalling molecules for the proper biological and structural functioning of cells. Long-term studies on the metabolism of sphingolipids have provided evidence for their role in the pathogenesis of a number of diseases. As many inflammatory diseases, such as lysosomal storage disorders and some dermatologic diseases, including psoriasis, atopic dermatitis and ichthyoses, are associated with the altered composition and metabolism of sphingolipids, more studies precisely determining the responsibilities of these compounds for disease states are required to develop novel pharmacological treatment opportunities. It is worth emphasizing that knowledge from the study of inflammatory metabolic diseases and especially the possibility of their treatment may lead to insight into related metabolic pathways, including those involved in the formation of the epidermal barrier and providing new approaches towards workable therapies.

## 1. Introduction to Sphingolipids

Sphingolipids (SLs), first described by J.L.W. Thudichum in 1884 and named after the sphinx in Greek mythology, were thought to be part of the dangerous riddle of the brain. For approximately 100 years, sphingolipids functioned solely as structural components of cellular membranes. SLs compose a class of lipids with a polar head group and two nonpolar tails. Mammalian sphingolipids contain a 14- to 26-carbon chain sphingoid backbone attached to a head group and are conjugated to an acyl group of varying carbon chain length [1]. The variety of SLs is achieved by the incorporation of fatty acids that differ in chain length, while the substitution of different head groups yields complex sphingolipids. Replacing the hydrogen head group with phosphocholine yields sphingomyelin (SM), whereas substitution with sugars leads to the generation of glycosphingolipids (GSLs). The glycolipids include cerebrosides that have only one sugar attached (either glucose or galactose), globosides that have either a di-, tri-, or tetra-saccharide but do not contain sialic acid, sulfatides that contain a sulphate group and gangliosides that have complex oligosaccharides attached and include at least one sialic acid residue. Sphingomyelins are the only sphingolipid class that are also phospholipids. They comprise nearly 85% of all sphingolipids, particularly the myelin sheath and 10–20% of the total plasma membrane lipids found in humans. Substitutions of ceramides with different fatty acid chain lengths yields a variety of sphingomyelin species such as C16-SM, C18-SM and C20-SM. Cerebrosides is the common name for monoglycosylceramides (galactosylceramide and glucosylceramide), glycosphingolipids which are important components of animal muscle and nerve cell membranes. Sulfatides, or sulphated galactocerebrosides were the first SLs isolated from the human brain by Thudichum (1884). They are found at high concentrations in the brain, kidney and spleen [2,3]. In the nervous system, sulfatides are especially enriched in myelin, composing more than 70% of lipid constituents and almost 30% of those lipids are comprised of sulfatide and its non-sulphated precursor galactocerebrosides [4]. Globosides are major glycosphingolipids of human erythrocyte membranes [5] and have attracted particular interest, as they are important for cellular interactions including blood-type determinants. In the late 1930s, Ernst Klenk detected a new group of acid glycosphingolipids as storage material in the brain of patients suffering from Tay-Sachs disease, a lysosomal disorder [6]. Due to the abundance in normal ganglion cells, these were therefore named gangliosides. They are sialic acid-containing glycosphingolipids, which occur especially on the cellular surfaces of neuronal cells, where they form a complex pattern and contribute 10–12% of the lipid content [7]. The region mostly abundant with gangliosides is the brain. In the grey matter their occurrence is more or less 5 times higher than in the white matter. Process of gangliosides synthesis starts in endoplasmic reticulum and is continued by modifications in Golgi apparatus, including addition of carbohydrate moieties to lipid molecules. The main core of most gangliosides, excluding GM4 derived from galactosylceramide (GalCer), is lactosylceramide (LacCer). First addition of sialic acid to LacCer results with the simplest ganglioside, GM3, following additions yield GD3 and GT3 derived from GM3 and GD3, respectively. More complex gangliosides, belonging to the a, b and c series are derived from GM3, GD3 and GT3 by the multistage modifications. Brain development is accompanied with changes in ganglioside pattern from prevalence of simple to complex ones [8]. Additionally, glycolipid composition has also been found to be diverse between singular neuronal cell types [9]. Human and rodent embryonic brains are abundant with GM3 and GD3 gangliosides while during development, the expression of these simple gangliosides drags down with parallel increase in expression of more complex ones, like GM1, GD1a, GD1b and GT1b. This conversion can be associated with reversal in expression levels of ganglioside synthases.

The past two decades have seen significant advances in sphingolipid research which, besides being major membrane building blocks of eukaryotic cells, serve as bioactive signalling components and therefore carry out a multitude of critical cellular functions. When cellular signalling mechanisms regulated by sphingolipids were recognized as critical players in metabolic diseases in 2010, the first sphingolipid receptor modulator was employed as a therapeutic [10]. Progress in this area may contribute to a better understanding and therapeutic options for complex diseases such as atherosclerosis, diabetes, metabolic syndromes and cancer. Here, we will review the complexity of sphingolipid signalling in the field of metabolic and inflammatory-related disorders.

## 2. Sphingolipid Metabolism and Its Role in Cellular Processes

Sphingolipids can be basically defined as lipid molecules with the sphingoid core. This sphingoid backbone is acquired due to condensation of amino acid (principally serine) and a fatty acid (principally palmitate) [11]. The most common SLs are ceramide (Cer) and sphingosine (Sph). They differ in structure with the presence or absence of an acyl chain. Further modification by phosphorylation of the 1-hydroxy group generates ceramide-1-phosphate (C1P) or sphingosine-1-phosphate (S1P), respectively. Versatile head groups at this position are characteristic for other sphingolipids, like sphingomyelin with the phosphorylcholine head group, glucosylceramide (GluCer) and galactosylceramide (GalCer) with a single sugar molecule linked to ceramide.

**Figure 1:**
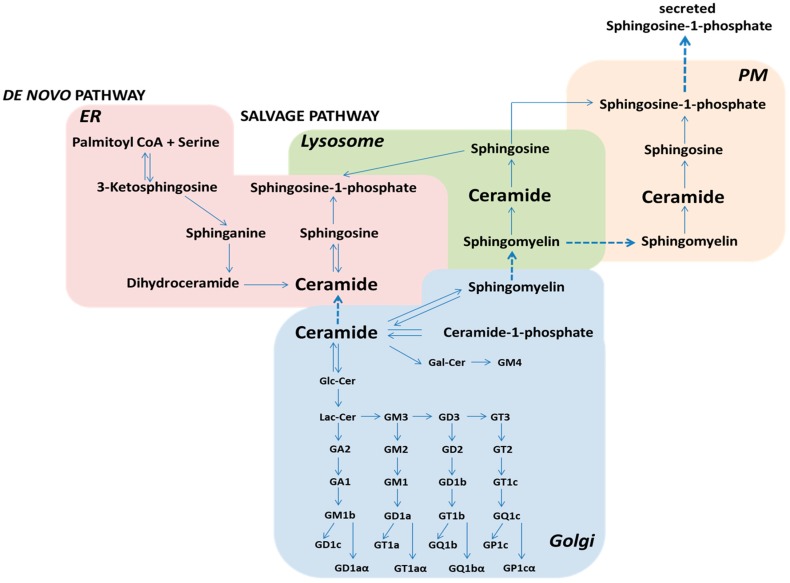
Overview of sphingolipid metabolism.

Ceramide is the primary unit of a number of complex sphingolipids, containing a sphingosine backbone, a fatty acid side chain and a hydrogen atom as the head group, including glycosphingolipids, sphingomyelin and ceramide-1-phosphate [11,12,13]. The de novo synthesis of SLs involves a single biosynthetic pathway, with the end product being ceramide [12]. From Cer as a central point, SL metabolism branches out in four main directions, with three altering the ceramide head groups (Figure 1). These include the phosphorylation of Cer by ceramide kinase (CerK) to produce C1P [14], the addition of phosphocholine by sphingomyelin synthase (SMS) to produce SM [15] and the addition of a sugar molecule by glucosyl- and galactosyl-ceramide synthases to create GluCer or GalCer, respectively [11].

All eukaryotic cells have the capacity to produce sphingolipids via a de novo pathway in the endoplasmic reticulum (ER) [12]. The first biosynthetic steps leading to the formation of ceramide are controlled by membrane-bound enzymes at the cytosolic face of the ER [16]. The formation of lactosylceramide, which is followed by modification of glycosphingolipids and sphingomyelin, occurs on the luminal face of the Golgi membranes [17,18]. The entry point is the condensation of serine and palmitoyl-CoA by the rate-limiting enzyme serine palmitoyl transferase (SPT). The product of the reaction—dihydrosphingosine (sphinganine)—is further reduced, acylated and desaturated to generate ceramide, a key intermediate. Alternatively, ceramide can be generated from the hydrolysis of complex sphingolipids. For example, the cleavage of sphingomyelin by sphingomyelinases results in phosphatidylcholine and ceramide. The heterogeneities within the ceramide part result from its biosynthesis at the endoplasmic reticulum, while the diversity of cell surface glycans, including gangliosides, is generated within the Golgi complex [19]. Once incorporated into complex sphingolipids within cellular membranes, ceramide can be liberated by hydrolysis but its range of activity is locally restricted by its hydrophobic nature. Only sphingosine, as a soluble substance, can be recycled via the salvage pathway to regenerate ceramide into other cellular compartments. As ceramide is considered to be the hub of sphingolipid metabolism, enzymes involved in this process function to maintain homeostasis. The control of sphingolipid synthesis by cells is regulated by substrate availability, as well as by other mechanisms related to lipid composition and membrane equilibrium [12,20]. The availability of the substrate palmitoyl-CoA enhances the de novo synthesis of sphingolipids. Since free palmitate levels rise in obesity and metabolic excess, sphingolipid flux through this pathway is enhanced [21,22]. The metabolic network of sphingolipids is connected through a series of chemical reactions that allows the production of distant metabolites from other ones [23]. Reactions between entry and exit points of sphingolipid metabolism are inter-convertible [1] and constant flux with the balance between metabolites determining cellular fate is sustained. For instance, sphingosine is mostly generated by the lysosomal acid ceramidase, released from the lysosome and used in the ER. About 70–90% of newly formed sphingolipids in differentiated cells, e.g., neurons, is generated by this salvage pathway, for the biosynthesis of sphingolipids and sphingosine-1-phosphate [24,25,26]. This highlights the importance of lysosomal metabolism for the synthesis of cellular SLs.

## 3. Diseases Associated with Defects in Sphingolipid Metabolism

A defect in sphingolipid degradation underlies the pathomechanism of many diseases. The only known human disorder linked to abnormal SL biosynthesis is the human autosomal recessive infantile-onset symptomatic epilepsy syndrome (salt and pepper developmental regression syndrome; SPDRS) (OMIM 609056), which is a defect in ganglioside biosynthesis, caused by a nonsense mutation in the gene encoding sialyltransferase (GM3 synthase) (EC 2.4.99.9) [27]. Malfunctions in the enzymatic degradation of glycosphingolipids have been linked to a family of devastating, incurable genetic disorders belonging to the family of lysosomal storage diseases (LSDs).

### 3.1. Defect of Ganglioside Metabolism

Gangliosidoses are a group of inherited disorders caused by defects in ganglioside catabolism. They appear in almost all age groups with severe, versatile clinical symptoms mainly affecting central nervous system [28]. Patients present mostly early infantile and a late infantile or juvenile onset with fatal end to their disease.

#### 3.1.1. GM1 Gangliosidosis

GM1 gangliosidosis is an autosomal recessive lysosomal storage disorder caused by mutations in the *GLB1* gene, leading to deficient beta-galactosidase activity. Beta-galactosidase (EC 3.2.1.23) catalyses the removal of terminal beta-galactose residues from glycoconjugates including ganglioside GM1, oligosaccharide and keratan sulphate. The enzymatic defect results in the accumulation of these substrates in nervous tissues and visceral and skeletal tissues. The primary features are neurodegeneration and skeletal abnormalities. There are three main clinical variants categorized by the severity of symptoms, age of onset and variable residual enzymatic activity of beta-galactosidase.

The classic or infantile type (type 1) (OMIM 230500) is the most common severe degenerative encephalopathy, presenting between birth and 6 months with coarse facial features, skeletal dysostosis and hepatosplenomegaly. Oedema, failure to thrive and hypotonia are other characteristic findings. There is usually the rapid progression of symptoms with the development of spasticity, seizures and general neurological deterioration with death typically occurring by age 2.

Progressive cerebral deterioration is characteristic of the late infantile/juvenile form of GM1 gangliosidosis (type 2) (OMIM 230600) as well. However, symptoms usually start at around 1 year of age with the regression of gained skills and motor delay in the absence of dysmorphisms and organomegaly; later, mental deterioration and spastic, cerebellar and extrapyramidal signs dominate the neurological picture, probably as a consequence of the predominant basal ganglia storage of gangliosides. Seizure management is often a major issue and these patients usually die between ages 3 and 7 [29].

The chronic/adult subtype of GM1 (type 3) (OMIM 230650) is the least common subtype with the phenotype consisting of dystonia, progressive ataxia and cerebellar dysarthria. These patients do not have the typical dysmorphic facies. Type 3 has a slowly progressive course and predominant extrapyramidal features without visceral or skeletal changes [30].

#### 3.1.2. GM2 Gangliosidosis

The GM2 gangliosidoses are a group of lysosomal lipid storage disorders caused by mutations in at least 1 of 3 recessive genes: *HEXA*, *HEXB* and *GM2A*, associated with the excessive accumulation of GM2 in the brain, which leads to neurological symptoms. 

The defined products of the 3 genes are the alpha subunits of b-hexosaminidase A (Hex A; EC 3.2.1.52), the beta subunits of Hex A (EC 3.2.1.52) and the GM2 activator protein, respectively.

Appropriate catabolism of GM2 ganglioside requires proper function of products of all 3 genes. Deficiency in activity of any of these enzymes results in abnormal lysosomal accumulation of the substrate in neurons, leading to cell death. 

The GM2 gangliosidoses are rare autosomal recessive genetic disorders that include Tay-Sachs disease (TSD, OMIM 272800), Sandhoff disease (SD, OMIM 268800) and GM2 ganglioside activator protein (GM2A) deficiency (variant AB, OMIM 272750) [31].

#### 3.1.3. Tay-Sachs Disease

Tay-Sachs disease (TSD) and its variants are caused by the absence of or defects in the alpha subunit of Hex A. In TSD, massive neuronal accumulation of sphingolipid GM2 gangliosides and its asialo derivative GA2 results in the progressive loss of central nervous system function. TSD has autosomal recessive inheritance. The specific populations that are at a higher risk of TSD are: Ashkenazi Jewish (AJ), French Canadian, Irish, Pennsylvania Dutch and Cajun communities [32].

In the acute infantile variant, after a period of normal development, affected individuals experience slow neurological decline and death in infancy or early childhood.

Main symptoms of TSD are decreased attentiveness and activeness due to loss of motor skills and progressive weakness. Increased startle response begins between three and six months of age and is accompanied with progressive evidence of neurodegeneration, including seizures, blindness, spasticity and eventual incapacitation [31].

Other variants of HexA deficiency, like juvenile (subacute), chronic and adult-onset are characterized by later onset with slower progression with more variable neurological symptoms. Common are progressive dystonia, motor neuron disease and spinocerebellar degeneration, and, in some cases of adult-onset disease, a bipolar form of psychosis [33]. 

#### 3.1.4. Sandhoff Disease

Sandhoff disease is a rare, severe, autosomal recessive lysosomal storage disorder, which is caused by a mutation in the beta subunit of the hexosaminidase A and B enzymes, resulting in an accumulation of glycosphingolipids and oligosaccharides in both grey matter nuclei and myelin sheaths of the white matter. These catabolic enzymes are needed to degrade the neuronal membrane components, ganglioside GM2, its asialo derivative GA2 and the glycolipid globoside in visceral tissues [34]. Sandhoff disease is not as strongly linked to ethnic groups as TSD. It seems to be more frequent in the Creole population of northern Argentina, the Metis Indians in Saskatchewan, Canada and people from Lebanon [35]. It has three clinical subtypes (infantile, juvenile and adult forms) and represents around 7% of all lysosomal storage disorders [36]. The infantile form presents in the first 6–18 months of age with developmental delay, the regression of milestones and startle response, hypotonia and convulsions [37]. In the course of Sandhoff disease symptoms like seizures, vision and hearing loss, intellectual disability and paralysis occur. Additional feature is a cherry-red spot, an eye abnormality located in central retinal artery occlusion, characteristic for the macula of the eye in a variety of lipid storage disorders.

Some infants affected by Sandhoff disease have an additional peripheral disease consisting of hepatosplenomegaly and dysostosis multiplex [31]. Lifespan of the children with the severe infantile form of Sandhoff disease is usually diminished to early childhood. In case of other, uncommon forms of SD, signs of the disease are mostly milder than in infantile form and can be observed from childhood, adolescence, or even adulthood. In patients with later onset, or in adult cases, symptoms may be those of spinocerebellar ataxia or dystonia [35].

#### 3.1.5. GM2 Gangliosidosis AB Variant

GM2 gangliosidosis, AB variant, is an extremely rare (only a few cases have been reported worldwide) autosomal recessive inherited disorder caused by mutations in the *GM2A* gene which encodes the GM2 ganglioside activator protein (GM2AP) [28]. A small (~25 kDa) amphiphilic protein, GM2AP, is necessary for the solubilization of GM2 ganglioside in endolysosomes and its presentation to β-hexosaminidase A [38]. Due to phenotypic similarities, this form of GM2 gangliosidosis is hard to distinguish from the infantile form of Tay-Sachs disease [39].

#### 3.1.6. Ganglioside GM3 Synthase Deficiency

Ganglioside GM3 synthase (also called lactosylceramide alpha-2,3-sialyltransferase) deficiency (OMIM 609056) is caused by a mutation in the *ST3GAL5* gene and is a rare metabolic disorder inherited as an autosomal recessive trait. Ganglioside GM3 synthase (EC 2.4.99.9) is the key enzyme involved in the initial stages of the biosynthesis of complex ganglioside species. The enzyme deficiency causes the complete absence of GM3 and all downstream biosynthetic derivatives [27].

Symptoms of the disease begin within the first weeks or months of life and are characterized by infantile onset of refractory and recurrent seizures associated with profoundly delayed psychomotor development and/or developmental regression, as well as abnormal movements and visual loss [40]. Numerous types of seizures are probable, including generalized tonic-clonic seizures (also known as grand mal seizures), which are responsible for muscle rigidity, convulsions and loss of consciousness. Certain affected children also struggle with extended episodes of seizures called non-convulsive status epilepticus [41]. Affected individuals develop hypo- or hyper-pigmented skin macules on the trunk, face and extremities in early childhood [42]. Symptoms also include difficulty feeding, irritability and vomiting. GM3 synthase deficiency includes both cases described as Amish infantile epilepsy syndrome and cases described as salt & pepper syndrome.

### 3.2. Sialo-Oligosaccharides Accumulate in Sialidosis

Lysosomal sialidase (EC 3.2.1.18) has a dual physiological function; it participates in the intra-lysosomal catabolism of sialylated glycoconjugates and is involved in cellular immune response. Mutations in the sialidase gene *NEU1* result in an autosomal recessive disorder, sialidosis (OMIM 256550), which is characterized by the progressive lysosomal storage of sialylated glycopeptides and oligosaccharides [43,44]. Sialidoses are classified on the basis of their phenotype and onset age. Sialidosis type II (mucolipidosis I or lipomucopolysaccharidosis), with infantile onset, has a more severe phenotype characterized by coarse facial features, hepatomegaly, dysostosis multiplex, Hurler-like phenotype and developmental delay [45]. Sialidosis type I (Cherry red spot myoclonus syndrome) is a milder, late-onset, normosomatic form of the disorder. Type I patients develop visual defects, myoclonus syndrome, cherry-red macular spots, hyperreflexia and seizures. Individuals with the late and milder type develop visual impairment and ataxia in the second or third decade of life [44].

### 3.3. Accumulation of Globosides

Fabry disease (FD, OMIM 301500) is an X-chromosomal-linked lysosomal storage disorder with a recessive mode of inheritance. The disease is caused by a deficient α-galactosidase A enzyme (a-d-galactoside galactohydrolase, EC 3.2.1.22; a-Gal A), which results in the intracellular accumulation of neutral glycosphingolipids (predominantly globotriaosylceramide (Gal a 1–4Gal b 1–4Glc b 1-1Cer; Gb3; or ceramide trihexoside), leading to the dysfunction of many cell types and includes a systemic vasculopathy [46]. α-GAL A liberates galactose from globotriaosylceramide (Gb3) and related glycosphingolipids. The major organs affected are the kidneys, heart and nervous system [47]. The main clinical manifestations of FD are neuropathic pain, proteinuria and renal failure, left ventricular hypertrophy and stroke. Male Fabry patients present minor facial dysmorphic features, including, periorbital fullness, conspicuous supraorbital ridges, bushy eyebrows, pronounced nasal angle, shallow midface, full lips, prominent nasal bridge, broad alar base and posteriorly rotated ears [48]. The classic form (type 1), with an onset in childhood or adolescence, occurrs in males with less than 1% α-Gal A enzyme activity. For this form, periodic crises of severe pain in the extremities (acroparesthesia) are typical, along with the appearance of vascular cutaneous lesions (angiokeratomas), sweating anomalies (anhidrosis, hypohidrosis and rarely hyperhidrosis) and characteristic corneal and lenticular opacities. On the contrary, the type 2 (later-onset) phenotype is associated with residual a-Gal A activity, lacks Gb3 accumulation in the capillaries and small blood vessels and does not display the early manifestations of type 1 features (i.e., the acroparesthesias, hypohidrosis, angiokeratomas, corneal dystrophy, etc.) [49]. In case of heterozygous females, symptoms are milder and an outbreak appears later than in males. Seldom, they may be almost asymptomatic throughout a regular life span or may have symptoms as serious as those observed in males with the classic phenotype [50].

Fabry disease may occur at any age, in children and adults [51]. It is a progressive disorder with a decreased lifespan to 50–55 years for men and 70 years for women [52].

### 3.4. Accumulation of Glucocerebrosides

Gaucher disease (GD, OMIM 230800) is a rare, autosomal recessive genetic disorder caused by mutations in the GBA1 gene. This defect causes drastically decreased activity of the glucocerebrosidase (GCase, also called glucosylceramidase or acid β-glucosidase, EC 4.2.1.25) lysosomal enzyme, which hydrolyses glucosylceramide (GluCer) into ceramide and glucose [53]. The consequences of this deficiency are generally attributed to the accumulation of glucosylceramide, in macrophages, inducing their transformation into Gaucher cells. In very rare cases, GD is due to mutations in the *PSAP* gene, which codes for the activator protein—an essential cofactor for the lysosomal degradation of glucosylceramide by glucosylceramidase (saposin C) [54].

Gaucher disease is classically categorized phenotypically into 3 main subtypes: non-neuronopathic type 1, acute neuronopathic type 2 and subacute neuronopathic type 3 [55]. GD type 1 (90% of cases) is the chronic and non-neurological form associated with organomegaly (spleen, liver), bone abnormalities (pain, osteonecrosis, pathological fractures) and pancytopenia. Other features include ocular pingueculae or nodules and dermal hyperpigmentation [56]. Type 2, the most severe form of the disease, is accompanied by neurological disease and characterized by early onset, rapidly progressing brainstem dysfunction, associated with organomegaly and leading to death before the age of 2. Type 3 has been subdivided into three different forms depending on the severity of neurological signs. Individuals with GD type 3 may have an onset before the age of two but often have a more slowly progressive course, with survival into the third or fourth decade [57].

Gaucher disease is the most common autosomal recessive disease in the Ashkenazi (Eastern European) Jewish population with a carrier frequency of 6% compared to 0.7–0.8% of the non-Jewish population [58].

### 3.5. Accumulation of Galactocerebrosides

Krabbe disease (globoid cell leukodystrophy, KD; OMIM 245200) is an autosomal recessive demyelinating sphingolipidosis caused by a deficiency in the lysosomal enzyme β-galactocerebrosidase (GALC, EC 3.2.1.46). GALC is required for the hydrolysis of galactocerebroside—a major myelin lipid—and of galactosylsphingosine or psychosine [59]. As psychosine leads to arrest in myelination and participates in progressive demyelination, it is cytotoxic to oligodendrocytes and Schwann cells [60]. The accumulation of GalCer triggers the formation of the characteristic multinucleated macrophages known as globoid cells.

KD is typically divided into subtypes based on age at onset, with the earlier onset associated with a more rapid progression. Infantile KD, which accounts for about 90% of cases [61], can be further divided into early-infantile disease, in which symptoms appear by 6 months of age and late-infantile disease, in which symptoms typically appear between 7 and 12 months of age. Children with the infantile form appear to be healthy for the first few months of life but show extreme irritability, spasticity and developmental delay. Patients with early-infantile disease experience rapidly progressive neurologic deterioration, seizures, psychomotor regression, loss of vision and hearing and ultimately early death, generally by 2 or 3 years of age [62]. Children with late-infantile disease experience similar symptoms but typically survive longer. In the late-infantile group, irritability, psychomotor regression, stiffness, ataxia and loss of vision were the most common initial symptoms. Common initial symptoms in juvenile-onset KD are vision problems, muscle weakness, gait changes and a loss of developmental milestones [63]. Spastic paraparesis is a prominent feature of adult-onset disease [64].

### 3.6. Storage of Ceramides

Farber disease (OMIM 22800) is an ultra rare (about 80 individuals affected by this condition have been reported worldwide) progressive multisystemic neurodevelopmental storage disorder caused by a deficiency of the lysosomal enzyme acid ceramidase (*N*-acylsphingosine deacylase, EC 3.5.1.23; AC) due to mutations in the acid ceramidase gene (*ASAH1*) [65]. AC is the lipid hydrolase leading to the degradation of ceramide into sphingosine and free fatty acids inside lysosomes. Low activity of this enzyme results in the accumulation of fatty materials, mostly ceramides [65].

This disease generally manifests with three symptoms: painful, swollen and progressively deformed joints, nodules under the skin and progressive hoarseness. All are very painful and are responsible for progressive joint stiffness, restrictions in motion by contractures and finally to the distortion of joints. Other features may comprise poor weight gain, sporadic fever and respiratory problems [65].

### 3.7. Lysosomal Storage of Sphingomyelin

Niemann-Pick (NP) disease is caused by an abnormality in lysosomes, which are unable to degrade lipids—mostly sphingomyelin and cholesterol; as a result, the latter accumulate inside these organelles to form cellular inclusions [66]. The NPD group is now divided into two distinct entities: acid sphingomyelinase-deficient Niemann-Pick disease (ASM-deficient NPD) resulting from mutations in the *SMPD1* gene and encompassing type A (OMIM 257200) and type B (OMIM 607616), as well as intermediate forms [67]; and Niemann-Pick disease type C (NPC) and type D, resulting from mutations in either the NPC1 or the NPC2 gene, respectively [68].

The enzyme acid sphingomyelinase (ASM), catalyses the hydrolysis of sphingomyelin to ceramide and a deficiency of the enzyme leads to the storage of sphingomyelin in the cells, mostly in reticuloendothelial cells [69]. Common manifestations of types A and B are hepatosplenomegaly and the appearance of cherry-red spots in the retina, whereas neurodegeneration is only manifested in patients with NPA [70]. 

## 4. Knowledge Implementation about the Metabolism of Sphingolipids in Learning the Mechanism of Other Diseases

Research in recent years has shown that sphingolipids are essential signalling molecules for the proper functioning of cells. Long-term studies on the metabolism of sphingolipids have provided evidence for their role in the pathogenesis of a number of diseases. The vast majority of this information relates to the formation of the skin barrier. In human skin, they have both structural and biological functions [71].

### 4.1. Sphingolipids in Cell Signalling

Until the late 1970s, lipids were believed to perform only structural roles as components of cellular membranes. The milestone for a central role of lipids in signal transduction was manifested by the discovery of the phosphoinositide cycle [72,73]. Nowadays, it is known that sphingolipids encompass a broad range of lipid molecules that elicit a wide range of signalling properties and cellular functions with special focus on the disparate nature of ceramide and S1P [23]. Under normal cellular homeostasis, sphingolipid metabolism is thought to be critical for fundamental cellular events such as membrane homeostasis, endocytosis, cell movement, nutrient transport and protein synthesis [20,23]. By demonstrating the inhibition of protein kinase C (PKC) by sphingosine and the stimulation of a ceramide-activated protein kinase in response to tumour necrosis factor (TNF)-α [74,75], sphingolipids have been perceived as modulators of various protein kinases and phosphatases, receptors and ion transporters [23] and key regulators of a broad range of cellular processes, including cell growth, adhesion, migration, senescence, apoptosis and autophagy [23,76]. Ceramide is generally associated with growth arrest and apoptosis, whilst the role of S1P is to promote cell proliferation and survival. The antagonistic nature and dynamic balance of intracellular Cer and S1P has been proposed to determine cell fate. Due to the differential modulation of apoptotic and autophagic processes, the sphingolipid network has emerged as a novel molecular switch of these pathways. In addition, other sphingolipid metabolites, such as sphingosine, dihydroceramide and gangliosides, have also been implicated in the regulation of apoptosis and autophagy. A shift of sphingolipid metabolism towards the increased production of ceramide is anti-proliferative, while a shift towards S1P favours survival [77]. The interaction of sphingomyelin, glycosphingolipid and cholesterol drives the formation of plasma membrane rafts which have been shown to be involved in cell signalling, macromolecule sorting and membrane trafficking. Toll-like receptors, class A and B scavenger receptors and insulin receptors are located in these lipid domains.

In addition, extracellular stimuli such as cytokines, hormones and cell stress (X-ray, UV irradiation) perturb sphingolipid metabolism via effects on both de novo synthesis and degradation [78,79,80,81]. This results in increased ceramide levels and induces the aggregation of lipid domains and the formation of ceramide-rich macrodomains. Such domains are thought to influence membrane signalling events [82,83], since lipid rafts localize signalling proteins. The expression of diverse enzymes in GSL metabolic pathways is regulated in response to extra- and intracellular stimuli, leading to alterations in bioactive lipid levels [84]. Also, many cell stimuli, like TNF and interleukin (IL)-1, have been reported to modulate the function of more than one of these enzymes in a cell- or tissue-specific manner [85]. Thus, the coordinated regulation of GSL metabolic pathway enzymes in response to cellular stimuli alters the lipid flux through this network [84]. Furthermore, disruption of the ER- and Golgi-associated systems by unrelated pathways (e.g., mucopolysaccharide degradation) may increase levels of a particular GSL and cause secondary accumulations of others [86]. These secondary storage compounds contribute directly to complex metabolic events, leading to multiple, apparently unrelated substrate storage diseases.

S1P, a bioactive sphingolipid metabolite, has been a well-known critical signalling molecule of many physiological and pathophysiological processes for 20 years, including atherosclerosis, diabetes [87] and osteoporosis [88]. It was shown that S1P acts through a family of cell surface receptors, controls cell trafficking and is crucial for the migration of immune cells throughout the body. All of these features might be essential in disorders associated with inflammation, like cancer [89], Alzheimer’s disease, obesity and metabolic syndrome [90].

Ceramide was first recognized in the 1990s as a regulator of apoptosis [91] and cellular senescence [92] and has since emerged as a critical mediator of cell death. Its structure is composed of acids linked with the heterogeneous sphingoid base by an amide bond. Both components are heterogeneous: the sphingoid base may take the form of sphingosine (monounsaturated dihydroxy long chain base LCBs) or sphinganine (saturated dihydrosphingosine) and, less commonly, a phytosphingosine (C4-hydroxyl derivative of dihydrosphingosine) and 6-hydroxy-sphingosine (C6-hydroxyl derivative of sphingosine) forms. More than 28 specific enzymes are involved in ceramide metabolism. Additionally, more than 200 individual species of Cer with different head groups and/or acyl chain lengths [93]. They are generated within the various biochemical pathways and subcellular compartments to exert particular activities [13]. Some studies suggest a pro-apoptotic role of C18:0-ceramide and pro-survival function of C16:0-ceramide [94]; however, an additional study has proposed that long-chain ceramides (C16:0, C18:0, C20:0) are anti-proliferative and have been linked to triacylglycerol-induced apoptosis in macrophages [95] whereas very long chain ceramides (C24:0, C24:1) promote cell proliferation [96]. C18-ceramide may also contribute to amyloid protein-mediated apoptosis in neuronal cells in Alzheimer’s disease [97], while, in contrast, growth arrest may be more specifically associated with an increase in C24-ceramide [98]. Intracellular ceramide-target enzymes and signalling pathways have been determined and they include activation of the membrane-associated guanine nucleotide-binding protein (Ras)/extracellular signal-regulated kinase (ERK)-mitogen-activated protein kinase (MAPK) cascade [99], as well as protein kinase PKC [100], kinase suppressor of Ras/MAPK [101], the Stress-activated protein kinases (SAPK)/Jun amino-terminal kinases (JNK) signalling pathway [102], Raf-1 [103] and also protein phosphatases PP1 and PP2A [104] and JNK [102].

Gangliosides, like ceramide are crucial players in apoptotic and autophagic machineries. The GD3 ganglioside has been recognized to directly permeabilize mitochondria in vitro as well as to promote mitochondrial permeability, cytochrome c release and caspase activation in intact cells [105,106]. GD3 is commonly located in the plasma membrane and endosomal/Golgi network. Upon stimulation, like TNF-α treatment, GD3 promptly translocates from the plasma membrane to the endosomal compartment, where it co-localizes with Rab5-positive early endosomes and Rab7-positive late endosomes [107]. In addition to explicit mitochondrial effects, the gangliosides GD1a, GM1 and GD3 have been shown to suppress NF-κB translocation to the nucleus, thereby attenuating the transcriptional expression of cytoprotective genes and promoting apoptosis [108,109]. Furthermore, autophagic cell death caused by gangliosides has been observed to be dependent on the processes like generation of reactive oxygen species (ROS), the inhibition of protein kinase B (Akt)/mechanistic target of rapamycin (mTOR), the activation of ERK and the formation of lipid rafts [110]. Reports from GSL synthase-deficient mice explain that simple-to-complex GSLs are nonessential for early brain development [111,112] but have crucial role in brain maturation and maintenance [113,114,115].

### 4.2. Sphingolipids in Central Nervous System Inflammatory Disorders

Alterations in inflammatory processes, including the mobilization of innate and adaptive immune cells to the tissues and also the activation of cytokine networks, are regulated by sphingolipids. On the other hand, SL metabolism is affected in tissues as a response to these changes. Both naïve and activated adaptive immune cells trafficking is regulated by S1P signalling via the S1P receptor-1 (S1PR1) [90] and organizes the optimal magnitude and duration of both humoral and cellular immune responses. The regulatory T cells, which attenuate inflammatory and autoimmune responses, are regulated by the S1PR1/Akt/mTOR signalling axis [116].

Neurological diseases associated with inflammation are usually accompanied by the aberrant activation of glial cells, which are resident immunoeffectors in the central nervous system (CNS) [117]. Upon activation, microglia and astrocytes change their morphology, immunophenotype and expression pattern of inflammatory mediators, including cytokines and chemokines [118]. Inflammation also occurs in visceral and CNS tissues as a general pathological event in glycosphingolipid-derived LSDs. As in neurological disorders, accumulated lipids are causally linked to a pro-inflammatory response, including activation of the microglial/macrophage system to produce inflammatory cytokines, leading to CNS damage.

In Gaucher disease type 1, glucosylceramide mainly accumulates in cells of mononuclear phagocyte origin. Therefore, serum levels of macrophage-derived cytokines IL-1, IL-1 receptor antagonist, IL-6, TNF-α and soluble IL-2 receptor, with both pro- and anti-inflammatory activities, have been variously elevated [55,119,120,121,122]. It can clearly be assumed that such mediators could play a role in disease progression [123,124,125]. Gangliosides activate microglia to produce the pro-inflammatory mediators—nitric oxide and TNF-α [126]—and also suppress toll-like receptor-induced pro-inflammatory cytokine expression [127]. For this reason, in humans and mice with Sandhoff disease, macrophage/microglial activation and subsequent cytokine expression result in neuronal dysfunction [128]. Furthermore, progressive CNS inflammatory alterations were parallel with the outbreak of clinical symptoms in Tay-Sachs, Sandhoff and GM1 gangliosidosis mice [129]. Disease progression was slowed by the anti-inflammatory drugs in several models of LSDs with glycosphingolipid metabolism disability [129,130]. Non-steroidal anti-inflammatory drugs (NSAIDs) (e.g., indomethacin, aspirin and ibuprofen) were even found to extend the lifespans of Sandhoff disease and NPC1 mice [129,131]. These studies confirm the pro-inflammatory reactions in the propagation and potential initiation of GSL-mediated CNS degeneration. Also, sulfatide partakes in assorted cellular events of the CNS and its cellular level has been linked to numerous inflammation-associated neuronal disorders. It was reported that sulfatide alone can trigger pathological inflammatory responses with changes in the morphology of primary microglia to their activated form. Reports about the role of sulfatide in driving pathological inflammatory responses indicate significant induction of heterogeneous inflammatory mediators in primary microglia and astrocytes leading to changes in the morphology of primary microglia to their activated form [132]. 

Sulfatide has been particularly involved in the pathogenesis and aetiology of chronic inflammatory diseases of the CNS, including multiple sclerosis (MS), which are described by inflammation and tissue damage accompanying myelin destruction [133,134,135]. 

It was also reported that sulfatide content in post-mortem brain samples from Parkinson’s disease patients is elevated by 30–40% in the superior frontal and cerebellar grey matter [136]. However, it seems more complicated, as sulfatide from lipid rafts of human brain grey matter is reduced by 30% in incidental Parkinson’s but not in classic Parkinson’s disease. Incidental form is defined as Lewy body pathology in the brain stem, while classic Parkinson’s is demonstrated as a complex motor disorder [137].

Moreover, sulfatide is dramatically depleted in Alzheimer’s disease [133,136,138]. In cerebral grey matter more than 90% reduction was observed compared to about 50% in the white matter. 

### 4.3. SL Alterations in Metabolic Syndrome

Sphingolipid alterations occur in obesity and metabolic diseases and influence major biological processes such as insulin sensitivity, lipid metabolism, inflammation and immune reactions. Increased levels of sphingolipids, in particular, ceramide, are then observed. Ceramide rather than sphingosine is responsible for fatty acid-mediated attenuation of insulin-stimulated glucose uptake and Akt phosphorylation [139]. This points to the crucial role of ceramide in the deregulation of glucose metabolism and is associated with resistance to insulin signalling [140,141]. Inhibition of the insulin receptor by the high glycosphingolipid content in the plasma membrane underlies this malfunction. Furthermore, the ganglioside GM3 seems to inhibit insulin receptor downstream signalling due to direct connection with the insulin receptor. Metabolic syndrome is characterized by aberrant lipid accumulation in non-adipogenic tissues such as the liver, muscle, heart and pancreas [21]. Increased sphingolipid levels play profound pathophysiological roles and its metabolism is also tightly connected with other lipid metabolic pathways and sterol metabolism [142]. Alterations in plasma membrane sphingomyelin results in variations in cellular cholesterol balance and trafficking, supposedly due to the affinity of these two lipid types.

### 4.4. SL Role in Skin Barrier Disorders

Sphingolipids play an essential role in maintaining normal skin function, with ceramide comprising about 50% of total epidermal lipids [143]. Ceramides are required for the permeability barrier, located in the horny layer of the epidermis. This barrier consists of the cornified envelope, a thick layer of terminal differentiated keratinocytes (corneocytes) [144,145,146], which are embedded in a highly organized multilamellar lipid matrix. This lipid-bound envelope consists of free fatty acids, cholesterol and ceramides [146]. Skin ceramides are at least partially generated from glucosylceramide degradation in the lamellar bodies [147]. The cellular distribution of GSLs can be altered in pathologic states. Mutagenized mice that are deficient in prosaposin or in β-glucocerebrosidase, both SL metabolizing enzymes, cannot process properly and fail to completely pass through the permeability barrier in the skin. They die soon after birth due to a permanent water loss. This rare observation also explains instant death of so-called Collodion babies [148] suffering from a complete loss of β-glucocerebrosidase. Due to sphingolipid disorders, many key steps for the correct formation of the extracellular lipid barrier aggregates have been resolved [149]. It is well known that patients suffering from atopic dermatitis possess a markedly reduced content of ceramide in the horny layer of the epidermis, which results in an impaired permeability barrier and leads to the characteristic dry and easily antigen-permeable skin [150]. Human keratinocyte development is accompanied by GluCer-synthase up-regulation at the transcriptional level [151], indicating the requirement of glucosylated ceramides for skin barrier formation. The need of sphingolipids for skin formation is one of the first proven physiological functions of sphingolipids. Covalent binding between ceramides and proteins like involucrin [152] (an important structural protein of the cornified cell envelope) can be expected to further increase the stability of the skin.

In recent years, research has shown that sphingolipids are essential signalling molecules for the proper functioning of cells. Long-term studies of the metabolism of sphingolipids have provided evidence of their role in the pathogenesis of numerous diseases. In human skin, they have both structural and biological functions. The vast majority of this information relates to the epidermal barrier formation [71].

Skin is the largest organ of the human body, acting as a first line of defence against the external environment. Its dynamic structure is not only a physical barrier but it is also a space for many biological reactions. The proper function of skin barrier requires many interactions between genetic and immunological factors that control the expression of proteins and enzymes controlling the sphingolipid metabolism [153].

Human skin is composed of three layers, with the deepest being the hypodermis, then the dermis and the most external being the epidermis. The hypodermis is a fatty tissue that is mainly responsible for thermoregulation and the storage of energy. The dermis is a dynamic structure of cells (especially fibroblasts), fibres and ground substances, which contains vascular and nerve plexuses [154]. The epidermis consists of four layers, starting from the outermost stratum corneum (SC), the stratum granulosum (SG), the stratum spinosum (SS) and the stratum basale (SB). The degree of keratinocyte differentiation varies depending on the layers of the epidermis. Keratinocytes in the stratum basale undergo rapid cell divisions, and, with the transition to the upper layers of the skin, they differentiate via the action of a higher concentration of calcium ions (Ca^2+^). The stratum corneum is composed of about 15 layers of tightly packed corneocytes, i.e., filled with dead keratin cells that are deprived of the nucleus and organelles. They are embedded in an extracellular lipid matrix with a unique pattern composed of 50% ceramides (CERs), 25% cholesterol (CHOL) and 15% free fatty acids (FFAs), with minor amounts of triacylglycerols (TAG), diacylglycerols (DAG) and cholesterol esters (CE) [155,156,157]. FFAs are composed of a single carbon chain, which is composed of 14 to 34 carbon atoms (C14–C34) in the epidermis, although it usually oscillates within the length of C24–C26. Most FFAs are saturated but monounsaturated fatty acids (MUFAs), polyunsaturated fatty acids (PUFAs) and hydroxyl-FFAs are also observed [158]. Beyond the structural function, they can act as signal transducers (e.g., as a ligand for peroxisome proliferator-activated receptors—PPARs), thereby leading to the differentiation of keratinocytes. Cellular metabolism and their proper level of hydration are controlled by cholesterol, occurring mostly in a sulphated form [153,159].

However, the most important group of compounds in the epidermis is ceramides, which belong to the family of sphingolipids. Except for the heterogeneous sphingoid base included in these compounds, the degree of differentiation of ceramides is also changed by the modifications of fatty acid molecules, such as the variability in chain length, degree of saturation and hydroxylation, or methylation pattern. FFAs, which are epidermal ceramides, are mainly found in non-hydroxy, α-hydroxy and ester-linked ω-hydroxy types [156,160].

Based on the Motta classification, 15 subclasses of ceramides were distinguished in the epidermis, of which omega (ω)-hydroxylated and ω-acetylated species are unique for this tissue [161,162]. Ceramides can be generated in several ways in the skin. First, they are de novo synthesized from palmitoyl-CoA and L-serine by serine palmitoyltransferase (SPT) in ER in the suprabasal layer of the epidermis. Then, they are converted to glucosylceramide (GluCer) or sphingomyelin (SM) and packaged into lamellar bodies. Lamellar bodies are organelles containing, apart from the above-mentioned lipid precursors, the enzymes necessary for their processing (such as glucocerebrosidase, acid sphingomyelinase or secretory phospholipase A2). In such forms, GluCer and SM are transported to the stratum granulosum and the stratum corneum, where they are converted back to free ceramide via β-glucocerebrosidase and sphingomyelinase, respectively. The proper function of hydrolysis enzymes is controlled by two types (C and D) of saposins (SAPs) [153]. The synthesis of ceramide increases along with the rate of epidermal cell differentiation, which is necessary for the integrity of the skin barrier [161]. Importantly, ceramides are biologically active metabolites, mediating a number of cellular pathways and, interestingly, their increased synthesis can also be affected by factors such as cellular stress. Through the activation of signalling pathways dependent on PKC-α and SAPK (c-jun N-terminal kinase, JNK), ceramides affect cell fate by inhibiting its proliferation and inducing apoptosis. Moreover, they regulate autophagy and ER stress. Ceramides are also invaluable for the proper functioning of epidermal cells by mediating processes such as cell-cell contact, adhesion and motility. Furthermore, they are essential factors for keratinocyte differentiation by the activation of apoptosis signal-regulating kinase 1 (ASK1) and caspase-14 [160,163].

Ceramides are not the only type of sphingolipid family in the skin tissue. One of the most prominent examples is S1P. This compound is formed during ceramide degradation via ceramidases and, subsequently, phosphorylated by sphingosine kinases to its active form. Vogler’s group indicates that the stimulation of in vitro cell culture of keratinocytes by S1P has an inhibitory effect on proliferation. Interestingly, this mechanism will not result in the induction of apoptosis but rather in cell cycle arrest through a reduction in the amount of cyclin D2 and the activation of p21 and p27 kinases [164,165]. An additional feature of S1P is its capacity to increase calcium ion levels, which are the most important signals for keratinocyte differentiation. In this way, S1P may contribute to the formation of corneocytes [166]. On the other hand, S1P is involved in the inflammatory response by the activation of TNF-α production. It results in the activation of the NF-κB pathway and, as a consequence, the increased secretion of IL-8 and endothelin-1 [167].

Lipids present in intercellular spaces are arranged in a specific, lamellar manner with a various repeat distance. The vast majority are densely packed in ordered (orthorhombic) structures [158]. Although lipids represent only 5–15% of the epidermis components (as compared to 75–80% protein content), they are extremely essential for the formation of a physical protection of the skin. Besides keratin and filaggrin, the ‘bricks in mortar’ structure (where the corneocytes are bricks and lipids are the mortar arranged in 25 layers) is the most important complex and creates the corneocyte lipid envelope (CLE). Structural proteins (involucrin, loricrin, trichohyalin) and intercellular lipids (mainly unusually long-chain, ω-acylated-hydroxyceramides) of CLE are generated in the stratum spinosum and are then transported to the granular layer of the skin. Here, they are covalently bonded and, simultaneously, loricrin is cross-linked with small proline-rich proteins (SPRs) by transglutaminases. The lipids from the Golgi apparatus are attached to the structure of the protein. The final stage occurs in the stratum corneum and includes further steps of cross-linking of the structural proteins, as well as the extrusion of fatty acids, cholesterol and ω-OH-ceramides to the surface of the cell membrane [157,168,169].

It is worth mentioning that epidermis is not only the layer of physical protection but performs many important functions, such as permeability (protection against water loss or control of the temperature), antimicrobial and antioxidant activity, or UV protection. Only impaired skin barrier integrity or the development of inflammation draws attention to the importance of this unique structure. With a better understanding of the mechanisms of lysosomal storage disorders (LSDs), it was possible to confirm a wide spectrum of sphingolipid functions. The aforementioned Gaucher’s disease provided indications for the association of sphingolipids with the epidermal barrier. The cause of Gaucher’s disease is a mutation in the gene encoding acid-β-glucosidase (GluCerase) leading to its absence or to a significant reduction of its synthesis. As mentioned above, this lysosomal hydrolase is essential for the formation of free ceramide by cleaving glucosylceramide (GluCer). The lack or decreased activity of GluCerase causes many symptoms, including hepatomegaly, splenomegaly and, in some cases also, neurological deficit [170]. Studies of patients with type 2 Gaucher’s disease have shown that there is a strong correlation between the deficiency of glucosylceramide and proper formation of the skin barrier; these observations were also confirmed in a mouse model of gene knockout, which demonstrated that homozygous animals had histological abnormalities in the epidermis. This is due to the fact that the majority of epidermal ceramide species are the origin of GluCer. These findings provide evidence that the activity of only a single enzyme, β-glucosidase, is crucial for generation of the protective function of the epidermis and that the ceramides are key intermediate compounds necessary for the proper progress of this process [161,171].

Noticeably, the above data can be reviewed on the basis of the results obtained for dermatological diseases, such as ichthyosis, atopic dermatitis and psoriasis. Psoriasis is a common, chronic, non-infectious skin disease. It is characterized by the excessive proliferation of keratinocytes accompanied by disturbances of the immune system, environmental factors and a genetic predisposition. Psoriasis can occur at any age but there are two main peaks of disease: type I (early) and type II (late). The early manifestation of the disease (usually at the age of 16–22 years) is associated with a positive family history, more severe course and numerous relapses. Overall, 80% of cases of this type are associated with the presence of the HLA-Cw6 locus. The second type of psoriasis is most common in people aged 57–60 years [172,173]. So far, several studies have identified 15 loci (PSORS1-PSORS15) on particular chromosomes, which are related to the susceptibility of developing psoriasis. This predisposition is dependent on at least several genes; however, the presence of alleles does not prejudge its occurrence [174,175].

On the basis of morphological changes, several forms of psoriasis have been distinguished. The most common (occurring in approx. 90% of patients) is the psoriasis vulgaris variant. A characteristic feature is the presence of reddened skin changes, with limited edges, increasing successively to the size of plaques [176]. This process occurs with the excessive angiogenesis and hyperplasia of the epidermis. Histologically, a significant or even complete loss of the granular layer of skin is also observed. On the other hand, the keratinocytes of the stratum corneum layer are characterized by very fast proliferation and incomplete differentiation, accompanied by parakeratosis. A somewhat different course is seen in pustular psoriasis, without the typical psoriatic plaques but with the formation of only 1–2 mm pustules. However, both forms may be present simultaneously (or undergo a mutual transformations), both in limited areas of the body or over its entire surface (erythrodermic form). In approx. 6–40% of patients, a chronic seronegative arthritis, i.e., psoriatic arthritis, may also develop [177,178].

Based on the recent studies, two major hypotheses explaining the process of pathogenesis have been proposed. According to the first, a major cause of psoriasis is the stimulation of keratinocytes by external stimuli. This is followed by the synthesis and secretion of pro-inflammatory cytokines and IFN-α, resulting in the activation of T cells. The overproduction of interleukins (such as IL-17A, IL-17F and IL-22) and the increased influx of neutrophils and T cells into the epidermis enhance the inflammatory response and cause the excessive proliferation of keratinocytes. This leads to reorganization of the tissue, degradation of the extracellular matrix and increased angiogenesis and thus the consolidation of inflammation and disease progression [179,180,181,182]. Much research is also devoted to the discovery of new therapeutic anti-psoriatic substances, which are tested in preclinical laboratory studies and clinical phase trials. The development of relevant in vitro and in vivo models of psoriasis is now a priority and an important step towards its cure [183].

**Figure 2:**
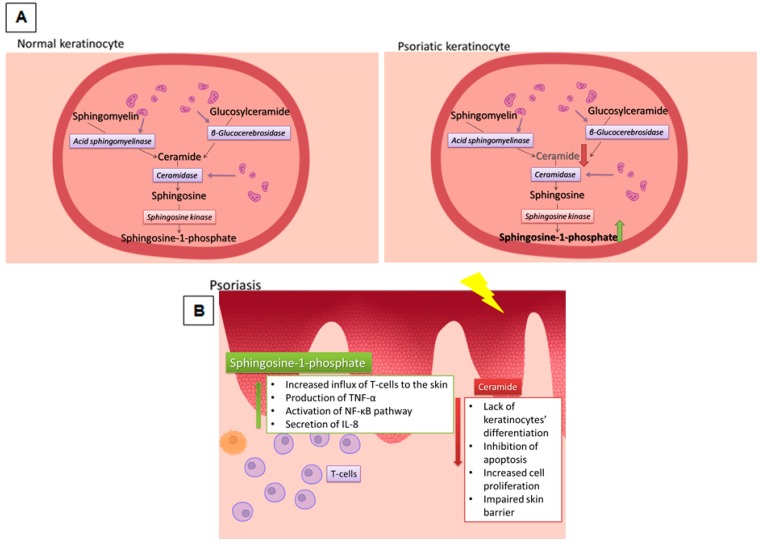
(**A**) Sphingolipid metabolic pathway in normal and psoriatic keratinocyte. In psoriatic keratinocytes, the level of ceramides is lowered, while the level of S1P is increased. (**B**) Possible effect of metabolic disturbances of sphingolipids on the development of psoriasis. Red arrow indicates a reduced level of ceramides, while green arrow presents an elevated level of S1P.

Although the term ‘psoriasis’ is most often defined as an immunological disorder in the literature, it is worth noting that the majority of pathogenesis is in fact concentrated on the skin barrier, which is confirmed by the analysis of SNPs in the genes encoding cornified envelope proteins [175]. Moreover, in lesional psoriatic skin, a depletion or quantitative changes of particular types of ceramides are observed. These changes are additionally enhanced by quantitative and qualitative disturbances of the extracellular matrix lipids. Studies of patients with psoriasis have shown a significant reduction in the synthesis of ceramide in lesional skin, which can contribute to increased transepidermal water loss (TEWL) [184]. However, the qualitative differences in the composition of ceramide species in the epidermis can affect the epidermal barrier dysfunction. Motta et al. showed strong quantitative differences between ceramides’ types in psoriatic lesion, i.e., 60% of them are decreased, while the remaining 30% are increased [162]. Moreover, SPT-cKO mice, with knockout of the gene encoding serine palmitoyltransferase (SPT), an enzyme necessary for the synthesis of ceramide, developed ‘psoriasis-like’ skin lesions with elevated levels of γδ T cells and pro-inflammatory factors, such as: β-defensins, IL-17A, IL-17F, IL-22, S100A8 and S100A9 [185]. Alterations are also observed for another enzyme, acid-β-glucosidase (GluCerase). The mRNA expression of GluCerase in skin punch biopsies is decreased in psoriatic plaques, but, interestingly, its level is even lower in non-lesional psoriatic skin compared to the control. The GluCerase activity is regulated by saposin (SAP). The mRNA and protein levels of SAPs were measured in the biopsies of skin tissue, and, in both cases, the significant reduction was observed (compared with the normal skin) [186,187]. The reduction of ceramide synthesis and the malfunction of enzymes necessary for its proper formation may be correlated with the severity of disease [184]. These changes are accompanied by a reduction in the signalling molecules protein kinase C-alpha (PKC-α) and c-jun N-terminal kinase (JNK), which are essential factors for the activation of apoptosis [163]. In contrast, the level of another sphingolipid, sphingosine, is markedly higher in psoriatic epidermis compared to non-lesional skin, what can be associated with positive correlation between changes of ceramidase level (an enzyme necessary for degradation of ceramide and sphingosine formation) and Psoriasis Area and Severity Index (PASI) score. These data are supported by evidence of increased circulating amounts of S1P form in severe psoriasis patients. S1P is an essential factor for T-cell migration and its increased quantity may cause intensified infiltration into the skin, exacerbating the immune response [188,189,190]. In keratinocytes, S1P activates the NF-κB pathway, which results in the increased production of cathelicidin antimicrobial peptides (CAMPs). CAMPs are defined as effectors of the innate immune response, with antimicrobial properties. One of them, LL37, is strongly associated with psoriasis. It activates monocytes and dendritic cells and indirectly affects the production of chemokines and cytokines such as IL-6 or IL-8. S1P prevents Cer-induced apoptosis in keratinocytes in response to ER stress. The lack of programmed cell death and the activation of an early immune response are characteristic features of psoriasis, which, as revealed, can be dependent on the balance between Cer and S1P (Figure 2) [191,192,193].

As can be deduced from provided data, ceramides are not only structural compounds that protect the skin from excessive water loss or environmental factors, they are an important link between many cellular processes, mediating programmed cell death (PCD) or controlling the proliferation rate. The diversity of functions that ceramides play in many types of cells means that the aberration of its metabolism may be the cause of diseases with different phenotypes, severities and courses.

## 5. Flavonoids, Compounds Modulating Sphingolipid Metabolism Used in Lysosomal Storage Diseases and Skin Disorders

Increasingly, it turns out that daily products which create our diet have a great impact on human health and disease treatment. Examples of bioactive plant-derived metabolites are flavonoids, which have been studied as potential agents in medicine for many years [194,195]. Flavonoids are ubiquitous plant secondary products, best known as the characteristic red, blue and purple pigments of plant tissues, which are essential part of daily dietary sources, like fruits and vegetables; however, they are also plentiful in tea, cocoa and wine. In higher plants, flavonoids are involved in UV filtration, symbiotic nitrogen fixation and floral pigmentation. They consist of over 8000 already-identified compounds that have a common chemical structure and may be further divided on the basis of their molecular structure into subclasses like flavanols, flavanones, favonols, flavones and isoflavones. Over the past decade, researchers have become fascinated in respect to various dietary flavonoids and therefore tried to elucidate some of the health benefits accompanying fruit- and vegetable-rich diets. The best-described property of almost every group of flavonoids is the capacity to act as antioxidants and anti-inflammatory factors [196]. This group consists of compounds that are considered to be therapeutic molecules for various inflammatory diseases, among them lysosomal storage diseases and skin disorders [197,198,199,200,201,202]. Thus, defining molecular bases of their biological actions and functions is essential to evaluate safety of these composites and to develop finest therapeutic procedures. Rather than their antioxidant activity, the ability of these compounds to modulate cell-signalling pathways appear to be essential among many of the biological effects of flavonoids. Genistein (isoflavone), kaempferol (flavonol) and daidzein (isoflavone) were previously found to be able to reduce the efficiency of glycosaminoglycan (GAG) synthesis in cells of patients suffering from mucopolysaccharidoses (MPS), inherited metabolic diseases that often manifest with brain disease symptoms [203]. It is worth mentioning that these flavonoids can cross the blood-brain barrier (BBB), which makes sense considering that these compounds are potentially useful in the optimization of treatment for neuronopathic forms of certain diseases. As flavonoids can cross the BBB, considering them as potentially useful compounds in the optimization of substrate reduction therapy for neuronopathic forms of LSDs (such as MPSs and SLs) appeared reasonable.

The latest findings of our group allowed us to learn about a putative flavonoid targetome which is responsible for significant molecular processes, such as an impairment of the production and enhancement of degradation of glycosaminoglycans and sphingolipids [204,205,206], or cell cycle and DNA replication regulation [207]. Transcriptomics analyses indicated that selected flavonoids influence the expression of several genes involved in cellular metabolism. The model of genistein-directed lysosomal biogenesis modulation was described for the first time, demonstrating directly that this naturally occurring compound alters the expression of genes involved in lysosomal metabolism [204]. Although the gene expression changes observed here were rather subtle, they appeared to be relevant in cellular GAG level normalization. By monitoring particular mRNA levels in human fibroblasts and mouse embryonic fibroblasts, it was found that genistein also stimulates the expression of *TFEB*, which was previously demonstrated to act as a master positive regulator of lysosomal biogenesis [204]. In the course of our work, we found that among the tested flavonoids, genistein, kaempferol and a mixture of these two agents manifested the most marked effects on the regulation of gene expression [206]. Remarkably, employment of genistein and kaempferol combination had more significant impact that those exposed by any of these flavonoids used alone. As these natural compounds can cross the BBB, it was reasonable considering them as potentially valuable in the treatment for neuronopathic forms of LSDs, including sphingolipidoses, in which accumulation of sphingolipids, especially glycosphingolipids, occurs. Indeed, in our studies we showed that flavonoids also have a significant impact on the expression pattern of dozens of genes involved in sphingolipid metabolism [205]. The transcriptomic analyses showed that the flavonol kaempferol, the isoflavone genistein and a combination of these two agents profiled the activity of the greatest number of genes. When considering particular tested conditions, we realized that numerous of transcripts with altered expression (59 of the 121 genes) at least in one experimental design, belong to various regulation GSL metabolism modules, such as GSL biosynthesis—globo-, lacto-, neolacto- and ganglio- series, lactosylceramide biosynthesis, glycerolipid metabolism, ceramide biosynthesis, sphingosine biosynthesis, sphingosine degradation, other sphingolipid metabolism genes, intracellular trafficking or fusion GSL catabolic enzyme modification. Among them were both positively and negatively regulated genes coding for enzymes involved in the production and degradation of specific lipid derivatives. In addition, in each individual pathway for the metabolism of specific classes of GSLs, there were genes with stimulated and inhibited activity and encoding enzymes functioning in both opposite paths. One might predict that the inhibition of a crucial part in the production of certain GSL may cause impairment of the entire process, even if other steps are stimulated, according to the bottleneck mechanisms, i.e., limitation of the efficiency of the whole pathway by the slowest process. Such a hypothesis requires however further experimental verifications. Interestingly, 16 genes were associated with well-known sphingolipid disorders: *ARSA* (metachromatic leukodystrophy, MLD), *ASAH1* (Farber lipogranulomatosis), *CLN8* (neuronal ceroid lipofuscinosis, late-Infantile Neuronal Ceroid Lipofuscinosis variant, CLN8), *GALC* (Krabbe disease), *GBA1* (Gaucher disease; Lewy body dementia, LBD), *GLA* (Fabry disease), *GM2A* (Tay-Sachs disease AB variant), *HEXA* (Tay-Sachs disease), *HEXB* (Sandhoff disease), *NAGA* (Schindler/Kanzaki disease), *NEU1* (sialidosis, salactosialidosis, mucolipidosis I), *NPC1* (Niemann-Pick disease type C), *NPC2* (Niemann-Pick disease type C), *PPT1* (Neuronal ceroid lipofuscinosis, Infantile Neuronal Ceroid Lipofuscinosis, INCL, CLN1), *SMPD1* (Niemann-Pick disease NPD type A and B) and *SUMF1*(Multiple sulfatase deficiency, MSD). It is worth mentioning that flavonoids can cross the blood-brain barrier, which is reasonable when considering these compounds as potentially useful in the optimization of treatment for neuronopathic forms of sphingolipidoses. In light of problems crossing the BBB during medication, it appears crucial to look for potential drugs that could be used in pathological sphingolipid storage.

Flavonoids with anti-inflammatory activities are applied worldwide as alternative medicaments among others for psoriasis because of their perceived beneficial impact on the skin state [200,201]. Ito’s group investigated the topical application of Glyteer (GL, soybean) on a psoriatic model in mice [208]. In this study, they observed that GL inhibits epidermal weight and its protein amount on the hyperplastic response in tested animals. Also, topical genistein was found to decrease psoralen-ultraviolet A (PUVA)-induced skin thickening and greatly reduce cutaneous erythema and ulceration in a dose-dependent manner [209]. As a result, in patients with psoriasis, combining genistein with PUVA therapy potentiated the therapeutic response of the latter and protected against complications. This isoflavone has also attracted attention as a potent agent in the treatment of psoriasis, not only due to its anti-proliferative and immunosuppressive properties but also as a mediator modulating the expression of various genes, whose products are involved among others in different phases of inflammation and proliferation [199]. Lastly, we examined the effects of isoflavone genistein on activated, spontaneously immortalized human keratinocytes, a ‘psoriasis-like’ HaCaT cell line, to find new potential targets for therapy and/or to develop a tool for treatment. Our results suggested that the aberrant expression of genes contributing to the progress of psoriasis can be improved by the action of genistein; they also explain in detail the effects of this isoflavone on signalling cascades in the human epithelial cell line HaCaT. However, the explicit mechanism of flavonoid action, especially in human epithelial cells, is still not elucidated. Recent studies have demonstrated that genistein increases cellular levels of sphingosine-1-phosphate, which stimulates the production of the key epidermal antimicrobial peptide (AMP) and cathelicidin antimicrobial peptide (CAMP) [210,211]. Genistein increased the production of S1P (without changes in ceramide levels) by inhibiting the activity of S1P lyase, an enzyme catalysing S1P breakdown to phosphoethanolamine and hexadecenal, as well as increasing the expression of key enzymes, e.g., acidic/alkaline ceramidases and sphingosine kinase 1 (SPHK1), which convert ceramide to S1P. Taken together, it has been shown that genistein induces CAMP production via an ER-β→S1P→NF-κB→C/EBPα mechanism rather than a VDR-dependent mechanism, illuminating a new role for oestrogens in the regulation of epithelial innate immunity and indicating the potential additional profits of dietary isoflavone genistein in enhancing cutaneous antimicrobial defence. The discovery of the importance of sphingolipid metabolism for healthy skin highlights relevant translational research that is directed towards novel therapies; in addition, it has opened up a new and interesting field.

## 6. Conclusions

This chapter aimed to provide an abbreviated overview of our current understanding of sphingolipid metabolism dysregulation in diseased mammalian systems and also to highlight some areas of treatment opportunities that are inadequately explored and understood. We discussed the abnormal sphingolipid world in inflammatory diseases such as lysosomal storage diseases and skin disorders, pointing to natural flavonoid compounds as potential drugs for the treatment of these illnesses, in which errors in sphingolipid metabolism occur. Obviously, it is a healthy scientific practice to regularly reflect on the progress that has been achieved within a field to bring light to significant research areas; these undoubtedly comprise matters related to human diseases with an inflammatory background.
